# High frequency edge network for accurate cardiac structure segmentation

**DOI:** 10.1016/j.isci.2026.116739

**Published:** 2026-07-14

**Authors:** Shuai He, Hui Xiong, Wenmiao Wang, Jiaxin Zhang, Min Zhu, Haijian Guo, Wei Qin

**Affiliations:** 1 Department of Cardiovascular Surgery, Affiliated Hospital of Nantong University, Nantong, China; 2 Department of Thoracic Surgery, The Second Qilu Hospital of Shandong University, Jinan, China; 3 School of Artificial Intelligence and Electronic Information, Nantong Vocational University, Nantong, China

**Keywords:** Cardiac segmentation, High-frequency driven modeling, Boundary delineation, Transformer, Semantic-edge re-alignment

## Abstract

Accurate cardiac structure segmentation is intrinsically a boundary delineation problem, where discriminative anatomical cues are largely encoded in high frequency components. We develop High Frequency Edge Network (HF-EdgeNet), a high frequency driven encoder decoder framework that incorporates structural cues throughout cardiac magnetic resonance imaging (MRI) segmentation. Specifically, HF-EdgeNet uses the High Frequency Edge Transformer (HF-EdgeT) to inject high frequency guidance into self-attention, introduces the High Frequency Adaptive module (HF-Adapte) to compensate for high frequency degradation during down sampling, and designs the Semantic Edge Bridge (SEB) block for high frequency semantic re-alignment during decoding. Experiments on ACDC and M&Ms show improved average dice scores and favorable boundary related performance over strong segmentation baselines. These results support explicit high frequency modeling for boundary sensitive cardiac image segmentation.

## Highlights

• HF-EdgeNet integrates high-frequency cues throughout cardiac MRI segmentation
• HF-EdgeT injects high-frequency guidance into attention for boundary-aware features
• HF-Adapte and SEB preserve and re-align structural cues across scales
• HF-EdgeNet improves cardiac segmentation on ACDC and M&Ms benchmarks

## Teaser

Health sciences; Medicine; Medical specialty; Internal medicine; Cardiovascular medicine

## Introduction

Accurate segmentation of cardiac structures is a cornerstone of quantitative cardiac imaging and plays a critical role in a broad range of clinical workflows, such as ventricular volume and ejection fraction estimation,^1^^,^^2^ myocardial thickness measurement,^3^ chamber morphology analysis,^4^^,^^5^ and treatment planning for cardiovascular diseases.^6^^,^^7^ Because these downstream indices are highly sensitive to boundary precision, reliable delineation of anatomical contours is essential for ensuring measurement repeatability and diagnostic confidence across subjects and imaging protocols.^8^^,^^9^ Nevertheless, cardiac anatomy presents unique challenges for automated segmentation: structures can be thin-walled and tightly adjacent, exhibit large inter-subject variability, and often appear with weak or spatially varying contrast due to imaging artifacts, partial volume effects, and motion-related degradations.^10^^,^^11^ These factors make the problem particularly boundary-sensitive, as small contour errors may propagate into noticeable deviations in clinically relevant metrics.^12^^,^^13^

While recent deep learning models have achieved promising performance on cardiac segmentation,^9^^,^^10^^,^^14^ the intrinsic nature of this task is often simplified as a region-level classification problem.^12^^,^^15^ In practice, cardiac structure segmentation is fundamentally a boundary localization task,^8^^,^^16^ where discriminative cues that separate adjacent anatomical structures are predominantly encoded in high-frequency components,^17^^,^^18^ such as sharp intensity transitions,^19^ narrow edges, and local curvature variations. Particularly for thin myocardial walls and low-contrast cardiac boundaries, the contrast between neighboring tissues is subtle,^3^ and the semantic appearance of different structures can be highly similar, making low-frequency intensity statistics insufficient for precise delineation.^13^ Consequently, accurate segmentation critically depends on the faithful preservation and modeling of high-frequency structural information, rather than solely relying on global or regional semantic similarity. Recent boundary-enhanced medical image segmentation methods have also demonstrated the value of explicit boundary cues for weak or ambiguous contour delineation.^20^^,^^21^ Different from methods that mainly use boundary information as auxiliary constraints or edge-guided attention, High Frequency Edge Network (HF-EdgeNet) treats high-frequency structural cues as an intrinsic modeling factor throughout attention aggregation, down-sampling compensation, and semantic-edge re-alignment.

Existing cardiac segmentation methods have substantially improved representation learning through convolutional, transformer-based, and hybrid encoder-decoder designs. Early convolutional neural network (CNN)-based approaches, particularly U-Net variants, established the dominant paradigm for medical segmentation,^22^ while subsequent methods introduced multi-scale fusion, attention mechanisms, and self-configuring training strategies, such as nnU-Net,^10^ to improve robustness across datasets and imaging modalities. More recently, transformer-based and hybrid CNN-transformer architectures, including UNet Transformers (UNETR),^14^ nnFormer,^23^ TransUNet,^24^ and Swin-UNet,^25^ have demonstrated strong performance by enhancing long-range dependency modeling. Related efforts further explored hierarchical and self-supervised transformer designs for medical image segmentation.^26^^,^^27^ However, despite these advances, precise delineation of thin myocardial walls and weak-contrast anatomical boundaries remains challenging, indicating that stronger structure-sensitive modeling is still needed.

To alleviate boundary ambiguity and structural inconsistency, another line of research has incorporated structural priors into segmentation models. Boundary-aware and edge-guided methods introduce contour branches, edge supervision, or boundary losses to enhance sensitivity to anatomical contours,^28^ while other studies impose shape, topology, or distance-based constraints, such as signed distance maps,^29^ skeleton supervision,^30^ and morphological regularization,^31^ to encourage anatomically plausible predictions. At the same time, recent hybrid CNN-transformer segmentation models, such as TransUNet,^24^ UNETR,^14^ SegFormer,^32^ and Mixed Transformer U-Net,^33^ strengthen global representation learning, while lightweight or multi-scale attention designs, such as the boundary-aware lightweight transformer (BATFormer),^34^ DMSA-UNet with Dual Multi-Scale Attention (DMSA),^35^ multi-scale dynamic sparse attention UNet,^36^ and FCT-Net,^37^ further improve feature interaction efficiency and segmentation capability. Nevertheless, most existing methods still treat structural cues as auxiliary constraints imposed at the output, side-branch, or loss level, rather than as primary factors explicitly involved in feature representation learning.^14^^,^^28^ As a result, structural information remains weakly coupled with attention aggregation, feature abstraction, and decoder re-alignment. In addition, progressive down-sampling still attenuates fine structural details, and decoder-side recovery is usually achieved through implicit skip fusion without explicit semantic-boundary realignment.^25^^,^^34^ These limitations motivate us to investigate a more deeply integrated high-frequency-driven segmentation paradigm.

To address these challenges, we propose HF-EdgeNet, a unified high-frequency driven segmentation framework that explicitly incorporates high-frequency priors throughout the entire encoder-decoder pipeline for cardiac structure delineation. Instead of treating high-frequency cues as auxiliary details, HF-EdgeNet elevates them to a primary modeling factor for semantic representation learning, structural preservation, and decoding re-alignment. Specifically, a High-Frequency Edge Transformer (HF-EdgeT) injects high-frequency guidance into self-attention to construct boundary-aware semantic representations; an HF-Adapte module preserves and compensates high-frequency information before down-sampling; and a lightweight semantic-edge bridge (SEB) block performs explicit high-frequency-semantic re-alignment during decoding. Extensive experiments on multiple cardiac MRI datasets demonstrate that HF-EdgeNet consistently outperforms strong CNN- and transformer-based baselines, yielding improved segmentation accuracy, boundary fidelity, and structural consistency.

## Results

This section presents the main experimental results of HF-EdgeNet for cardiac structure segmentation. We first show that the proposed framework achieves consistently strong quantitative performance compared with state-of-the-art methods on automated cardiac diagnosis challenge (ACDC) and multi-center, multi-vendor and multi-disease cardiac segmentation (M&Ms). We then provide qualitative comparisons and feature visualizations to illustrate its advantages in boundary recovery and structural consistency. Finally, ablation studies and parameter analysis are reported to further examine the contribution of each component and the stability of the proposed design.

### Dataset

We evaluate the proposed HF-EdgeNet on two publicly available cardiac MRI benchmarks, namely the ACDC dataset^38^ and the M&Ms dataset.^39^ Both datasets are widely used for cardiac structure segmentation and present different sources of anatomical and imaging variability, making them suitable for evaluating the robustness of boundary-sensitive segmentation methods.

The ACDC dataset is a widely used benchmark for short-axis cine cardiac MRI segmentation, with expert annotations for the left ventricle (LV), right ventricle (RV), and myocardium (MYO).^38^ Following the standard ACDC protocol, we use it to evaluate cardiac structure segmentation under challenging boundary conditions, such as low contrast, thin MYO, and anatomical variability.

The M&Ms dataset is a multi-center, multi-vendor, and multi-disease cardiac MRI benchmark that introduces stronger appearance variations across scanners, acquisition conditions, and patient populations.^39^ Similar to ACDC, the segmentation targets include the LV, RV, and MYO. Compared with ACDC, M&Ms presents greater inter-domain heterogeneity and more complex intensity/style variations, which makes contour recovery and structural consistency more challenging. Therefore, introducing M&Ms as an additional benchmark helps evaluate whether the proposed high-frequency modeling framework remains effective on a second and more heterogeneous cardiac MRI dataset.

In our experiments, the ACDC dataset is divided according to the official fixed split provided with the dataset, including separate training, validation, and testing sets. For the M&Ms dataset, we follow the official labeled-data split/protocol and perform training, validation, and testing accordingly. Standard preprocessing procedures, including intensity normalization and spatial resampling, are applied to all volumes to ensure consistent resolution across subjects and datasets.

### Implementation details

All experiments are implemented using the PyTorch framework and conducted on an NVIDIA RTX 4090 GPU. The proposed HF-EdgeNet is trained using the Adam optimizer with an initial learning rate of 1 × 10^−3^ and a batch size of 4. The network is trained for 400 epochs in total. To facilitate stable optimization and effective multi-scale feature learning, the learning rate is reduced to 1 × 10^−4^ in the final 50 epochs, during which the parameters of the backbone are fixed and only the decoder and prediction head are updated. This training strategy helps improve feature fusion and prevents overfitting in the late training stage.

For data augmentation, random rotation, random scaling, random cropping, contrast adjustment, and gamma correction are applied during training to enhance model generalization. Following common practice in cardiac MRI segmentation, all patient-level 3D volumes in the ACDC dataset are decomposed into slice-level 2D images for training, while evaluation metrics are computed at the patient level to ensure a fair comparison. Unless otherwise specified, all hyperparameters are kept consistent across experiments.

### Quantitative comparison

We quantitatively compare HF-EdgeNet with state-of-the-art CNN- and transformer-based segmentation methods on two cardiac MRI benchmarks, namely ACDC and M&Ms, including TransUNet, SwinUNet, MT-Unet, nnU-Net, UNETR, BATFormer, and several recent attention-enhanced UNet variants. Tables 1 and 2 report the average dice score over all samples, dice scores for individual cardiac structures (RV, Myo, and LV), as well as precision, recall, and two boundary-aware geometric metrics, i.e., the 95th percentile Hausdorff distance (HD95) and average symmetric surface distance (ASSD).

**Table 1:** Quantitative comparison on the ACDC dataset

Method	Dice (%) on all samples				Precision	Recall	HD95	ASSD
	Avg.	RV	Myo	LV				
TransUNet^24^	89.98	87.89	87.50	94.57	92.57	88.08	1.58	0.27
SwinUNet^25^	89.24	87.13	86.27	94.31	91.53	87.51	2.14	0.37
MT-Unet^33^	90.07	88.30	87.04	94.86	90.15	90.59	2.23	0.46
nnU-Net^40^	90.00	87.89	86.90	95.21	92.08	86.42	2.16	0.44
UNETR^14^	88.46	87.71	84.71	92.97	88.30	89.37	2.90	0.45
BATFormer^34^	91.93	90.55	89.67	95.56	**92.33**	91.84	1.19	0.19
DMSA-Unet^35^	90.37	88.96	87.14	95.02	90.85	90.34	1.39	0.24
UNETVL^41^	90.80	88.68	88.56	95.15	89.38	**92.79**	1.69	0.32
MDSA-Unet^36^	90.88	89.15	88.07	95.41	91.73	90.46	1.19	0.21
FCTNet^37^	90.92	89.01	88.65	95.09	91.33	90.96	1.43	0.26
Ours	**92.14**	**90.83**	**89.84**	**95.74**	92.24	92.27	**1.02**	**0.16**

We report the average dice score over all samples, dice scores for individual cardiac structures (RV, Myo, and LV), as well as precision, recall, HD95, and ASSD. The best results are highlighted in bold.

**Table 2:** Quantitative comparison on the M&Ms dataset

Method	Dice (%) on all samples				Precision	Recall	HD95	ASSD
	Avg.	RV	Myo	LV				
TransUNet^24^	87.76	92.88	83.21	87.18	90.37	87.91	1.79	0.47
SwinUNet^25^	87.54	92.68	83.59	86.34	90.17	86.29	1.67	0.49
MT-Unet^33^	88.32	93.54	84.11	87.32	90.20	88.54	1.70	0.47
nnU-Net^40^	88.06	92.80	83.35	88.03	89.63	88.78	1.88	0.52
UNETR^14^	87.80	91.17	83.78	**88.44**	89.15	88.04	1.83	0.49
BATFormer^34^	88.27	93.25	83.85	87.72	89.04	89.53	1.84	0.51
DMSA-Unet^35^	88.15	93.41	83.74	87.29	88.71	90.32	**1.61**	0.45
UNETVL^41^	88.33	93.40	83.76	87.82	89.07	90.36	1.97	0.51
MDSA-Unet^36^	88.06	93.72	83.35	87.12	88.82	89.50	2.20	0.55
FCTNet^37^	86.02	91.62	80.82	85.61	88.80	90.31	1.93	0.50
Ours	**89.08**	**94.25**	**84.87**	88.13	**90.47**	**90.54**	1.71	**0.44**

We report the average dice score over all samples, dice scores for individual cardiac structures (RV, Myo, and LV), as well as precision, recall, HD95, and ASSD. The best results are highlighted in bold.

As shown in Table 1, HF-EdgeNet achieves the best overall performance on ACDC, obtaining an average dice score of 92.14%, which is 0.21 percentage points higher than the strongest competing method BATFormer (91.93%). More importantly, HF-EdgeNet consistently attains the best dice scores on all three cardiac substructures, including RV, Myo, and LV, indicating that the proposed framework improves not only global overlap but also structure-specific delineation. This advantage is particularly meaningful for challenging regions such as the MYO, where thin walls and weak contrast make boundary recovery difficult. In addition to region-overlap accuracy, HF-EdgeNet also achieves the best boundary-aware geometric performance on ACDC, with the lowest HD95 (1.02) and ASSD (0.16), demonstrating that the proposed high-frequency-driven design yields more accurate and structurally consistent contours.

Table 2 further validates the effectiveness of HF-EdgeNet on the M&Ms dataset. HF-EdgeNet achieves the best average dice score (89.08%) among all compared methods, outperforming U-Net Vision-LSTM (UNETVL) (88.33%) by 0.75 percentage points in terms of average dice. For class-wise dice, HF-EdgeNet obtains the best scores on RV and Myo, and achieves competitive performance on LV. In addition, HF-EdgeNet achieves the best precision, recall, and ASSD, while maintaining a competitive HD95. These results indicate strong overall segmentation accuracy and average boundary accuracy on this additional cardiac MRI benchmark.

To further assess the statistical reliability of the average dice improvements, we performed two-sided Wilcoxon signed-rank tests on paired test-case results. On ACDC, HF-EdgeNet improved the average dice from 91.93% to 92.14% compared with BATFormer, with a *p* value of 0.0559, indicating a positive but not statistically significant trend at the 0.05 level. On M&Ms, HF-EdgeNet improved the average dice from 88.33% to 89.08% compared with UNETVL, with a *p* value of 0.0347, demonstrating a statistically significant improvement. These results further support the consistent effectiveness of HF-EdgeNet across different cardiac MRI benchmarks.

Overall, the quantitative results on both datasets show that HF-EdgeNet consistently improves segmentation accuracy while maintaining strong geometric fidelity on challenging cardiac boundaries. This behavior is well aligned with the design motivation of the proposed method, where adaptive high-frequency structural cues are explicitly integrated into attention modeling, cross-scale preservation, and decoder-side semantic-boundary re-alignment.

### Qualitative results

**Figure 1:**
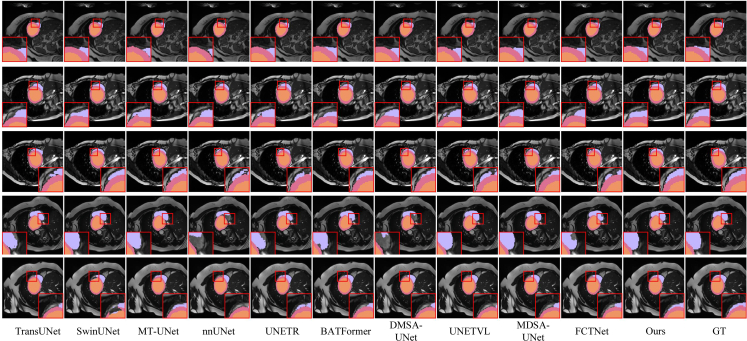
Qualitative comparison of segmentation results on the ACDC dataset Each row shows one representative case. From left to right are the predictions of different segmentation methods and the ground truth annotation. Red boxes indicate challenging boundary regions, and the corresponding local zoom-ins are provided to highlight contour details. This comparison illustrates the ability of different methods to delineate thin myocardial walls, weak-contrast boundaries, and locally ambiguous cardiac structures.

**Figure 2:**
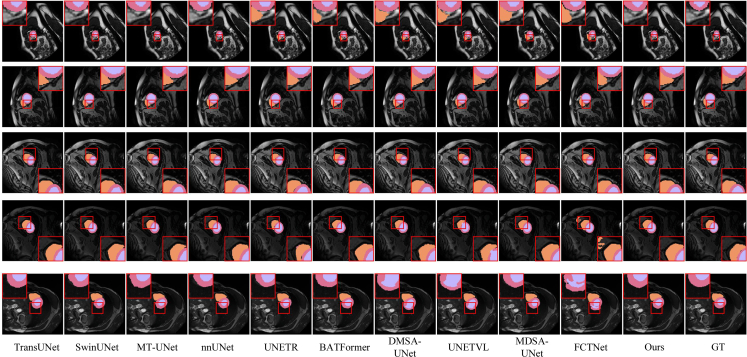
Qualitative comparison of segmentation results on the M&Ms dataset Each row shows one representative case. From left to right are the predictions of different segmentation methods and the ground truth annotation. Red boxes indicate challenging boundary regions, and the corresponding local zoom-ins are provided to highlight contour details. This comparison evaluates boundary delineation under stronger scanner-, vendor-, and disease-related appearance variations.

Figures 1 and 2 present qualitative comparisons between HF-EdgeNet and representative segmentation methods on the ACDC and M&Ms datasets, respectively. Overall, most competing approaches are able to produce visually plausible predictions for the major cardiac structures, especially in relatively clear and high-contrast regions. This observation is consistent with the quantitative results, indicating that recent CNN- and transformer-based segmentation models already provide strong baseline performance on both benchmarks.

Nevertheless, more noticeable differences emerge in challenging boundary regions, as highlighted by the red boxes and local zoom-ins. On the ACDC dataset (Figure 1), HF-EdgeNet generally produces smoother and more anatomically consistent contours around thin myocardial walls and weak-contrast transition areas. Compared with several competing methods, the proposed approach exhibits fewer local boundary irregularities, reduced contour drifting, and better preservation of slender structural details, yielding predictions that are more closely aligned with the ground-truth annotations in the enlarged regions.

A similar trend can be observed on the M&Ms dataset in Figure 2. Although this dataset presents additional appearance variations and more complex inter-case differences, HF-EdgeNet still shows more stable boundary delineation in the zoomed regions. In particular, the proposed method better preserves the continuity of the cardiac contours and reduces local distortions or jagged predictions that are more evident in several comparison methods. These observations are consistent with the improved dice and ASSD results reported in quantitative comparison, suggesting that the proposed framework achieves a better balance between region-level segmentation accuracy and boundary-level geometric consistency.

Overall, the qualitative comparisons on both datasets further support the central motivation of HF-EdgeNet. While many methods can recover comparable coarse semantic regions, explicitly modeling, preserving, and re-aligning high-frequency structural cues enables our method to produce more stable and boundary-consistent predictions, especially in thin, low-contrast, and anatomically complex regions.

### Visualization of high-frequency feature evolution

To better understand how HF-EdgeNet exploits high-frequency information for accurate boundary modeling, we visualize the spatial responses of high-frequency features at different network stages, as well as the boundary-enhanced representations produced by the SEB module.

**Figure 3:**
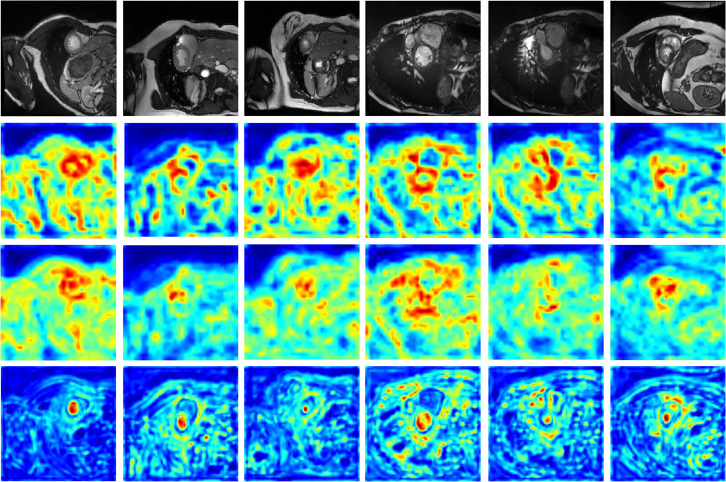
Visualization of high-frequency feature evolution in HF-EdgeNet on the ACDC dataset From top to bottom, the rows show input cardiac MR images, high-frequency responses at an early network stage (f3), high-frequency responses at a deeper stage (h3), and boundary-enhanced activations produced by the SEB module. The visualization shows how initially diffuse high-frequency responses are progressively refined into more compact and boundary-aligned structural activations during feature propagation and decoding.

Figure 3 illustrates representative examples from the ACDC dataset. From top to bottom, we present the input cardiac magnetic resonance (MR) images, the extracted high-frequency responses at an early stage (f3), the high-frequency responses at a deeper stage (h3), and the final boundary-enhanced activations generated by the SEB module. The high-frequency features are obtained through the learnable high-pass filtering operators embedded in HF-EdgeNet, which are designed to emphasize sharp intensity transitions and fine structural details.

As shown in the second row, the early stage high-frequency responses (f3) exhibit widespread activations over regions with rich texture and intensity variations, capturing abundant local edge and detail information. However, these responses are relatively diffuse and may include non-structural high-frequency noise. In contrast, the deeper high-frequency features (h3) shown in the third row become more spatially concentrated and semantically consistent, with stronger responses aligned along anatomically meaningful cardiac boundaries. This observation indicates that high-frequency information is progressively refined and structured as it propagates through deeper network layers.

The fourth row visualizes the outputs of the SEB module, which further enhances structurally consistent boundary responses while suppressing irrelevant high-frequency activations. Compared with the preceding high-frequency features, SEB produces cleaner and more compact boundary-aligned activations, demonstrating its effectiveness in selectively preserving boundary-related information during feature fusion and decoding. This progressive evolution from early high-frequency perception to boundary-focused enhancement qualitatively verifies that HF-EdgeNet learns boundary-aware representations in a data-driven manner.

### Ablation study

To comprehensively evaluate the design of HF-EdgeNet, we conduct a series of ablation studies on the ACDC dataset. In addition to the three core architectural components, namely HF-EdgeT, HF-Adapte, and SEB, we further investigate the effects of the adopted training strategy and the proposed frequency-aware supervision objective. All ablation experiments are conducted under the same training and evaluation settings for fair comparison.

#### Module-level ablation study

We first conduct a module-level ablation study to evaluate the contribution of each key component in HF-EdgeNet. Specifically, we progressively incorporate the proposed HF-EdgeT, HF-Adapte, and SEB block into a baseline encoder-decoder architecture and report the average dice score on the ACDC test set. The results are summarized in Table 3.

**Table 3:** Module-level ablation study on the ACDC dataset

Method	HF-EdgeT	HF-Adapte	SEB	Dice (%)
Baseline	×	×	×	90.16
+ HF-EdgeT	✓	×	×	90.98
+ HF-EdgeT + HF-Adapte	✓	✓	×	91.62
HF-EdgeNet (full)	✓	✓	✓	**92.14**

The best results are highlighted in bold.

As shown in Table 3, the baseline model without explicit high-frequency modeling achieves an average dice score of 90.16%. After introducing HF-EdgeT, the dice score increases to 90.98%, indicating that high-frequency guided attention effectively enhances boundary-aware semantic modeling. By injecting learnable high-frequency structures into the attention mechanism, HF-EdgeT enables the encoder to better capture fine-grained anatomical details that are critical for cardiac structure delineation.

Further incorporating HF-Adapte leads to an additional performance gain, improving the dice score to 91.62%. This improvement demonstrates that preserving high-frequency structural cues during down-sampling is essential for maintaining boundary integrity across scales. Without explicit compensation, high-frequency information tends to be progressively attenuated by resolution reduction, which limits the effectiveness of high-frequency guided attention in deeper layers.

Finally, integrating the proposed SEB block during decoding yields the best performance, achieving an average dice score of 92.14%. This result validates the necessity of explicitly re-aligning semantic representations with preserved high-frequency structural fields during up-sampling. By reconstructing boundary-consistent semantic features under high-frequency guidance, the SEB block effectively mitigates boundary drifting and improves the accuracy of fine structural recovery.

Overall, the progressive performance gains clearly demonstrate that HF-EdgeNet benefits from a closed-loop high-frequency driven design, where high-frequency cues are explicitly modeled, preserved, and re-aligned throughout the entire encoder-decoder pipeline.

#### Ablation on HF-EdgeT

We further investigate the effectiveness of the proposed high-frequency guided attention modeling by conducting an ablation study on different high-frequency injection strategies within HF-EdgeT. In this experiment, all other components, including HF-Adapte and SEB, are kept unchanged to isolate the impact of HF-EdgeT design.

Specifically, we compare three variants: (1) a vanilla transformer attention without any high-frequency modeling (no HF); (2) injecting high-frequency information to modulate the attention logits (HF on attention logits); and (3) injecting high-frequency information into the value branch, which corresponds to the proposed HF-EdgeT design (HF on value). The quantitative results are summarized in Table 4.

**Table 4:** Ablation study on different high-frequency injection strategies in HF-EdgeT

HF Injection Strategy	Description	Dice (%)
No HF	vanilla transformer attention	91.25
HF on attention logits	HF modulates attention weights	91.56
HF on value (ours)	HF injected into value branch	**92.14**

The best results are highlighted in bold.

As shown in Table 4, removing high-frequency modeling entirely leads to a noticeable performance drop, with the dice score decreasing to 91.25%. This result indicates that relying solely on region-level semantic similarity is insufficient for capturing fine-grained boundary structures in cardiac segmentation.

Introducing high-frequency cues by modulating the attention logits improves the dice score to 91.56%, suggesting that high-frequency information can bias the attention distribution toward boundary-related tokens. However, this strategy only influences the attention weights and does not explicitly enhance the aggregated feature content.

In contrast, injecting the high-frequency field directly into the value branch achieves the best performance, reaching a dice score of 92.14%. By explicitly enriching the value representations with boundary-sensitive structural information, the proposed HF-EdgeT enables high-frequency cues to be effectively propagated through the attention aggregation process. This design aligns well with the intrinsic role of the value branch in conveying detailed feature representations, leading to more accurate boundary-aware semantic modeling.

Overall, these results demonstrate that not only the presence of high-frequency information but also the manner in which it is integrated into the transformer attention mechanism plays a critical role in cardiac structure segmentation.

#### Ablation on SEB block

We further conduct an ablation study to evaluate the effectiveness of the proposed SEB block in the decoding stage. The SEB block is designed to explicitly re-align semantic representations with preserved high-frequency structural fields during up-sampling. In this experiment, HF-EdgeT and HF-Adapte are fixed, and only the design of the SEB block is varied.

Specifically, we consider three variants: (1) removing the SEB block entirely (no SEB), where the decoder relies solely on semantic feature propagation; (2) a one-way SEB variant (edge-to-semanti*c*), where high-frequency features are used to guide semantic reconstruction without semantic feedback; and (3) the full SEB block (bidirectional), which performs mutual refinement between semantic and high-frequency features. The quantitative results are reported in Table 5.

**Table 5:** Ablation study on different SEB block designs

SEB Variant	Description	Dice (%)
No SEB	semantic decoding only	91.62
Edge-to-semantic	one-way HF-guided semantic update	91.88
Bidirectional SEB (ours)	HF-semantic mutual re-alignment	**92.14**

The best results are highlighted in bold.

As shown in Table 5, removing the SEB block results in a dice score of 91.62%, indicating that semantic-only decoding is insufficient for fully recovering boundary-consistent representations, even when high-frequency information is preserved during encoding.

Introducing a one-way SEB design, where high-frequency features guide semantic reconstruction, improves the dice score to 91.88%. This result suggests that preserved high-frequency structural cues are effective in correcting boundary ambiguities during up-sampling. However, without semantic feedback, the high-frequency field may still contain spurious or noisy responses.

The full bidirectional SEB block achieves the best performance, reaching a dice score of 92.14%. By enabling mutual re-alignment between semantic and high-frequency representations, the proposed SEB block not only reconstructs boundary-consistent semantic fields but also refines high-frequency structures under semantic constraints. This bidirectional interaction effectively mitigates boundary drifting and enhances structural consistency in the decoding stage.

Overall, these results demonstrate that explicit HF-semantic re-alignment is essential for accurate boundary recovery, and that bidirectional interaction provides clear advantages over one-way refinement strategies.

#### Ablation on training strategy

We further conduct an ablation study to evaluate the effectiveness of the adopted late-stage training strategy for HF-EdgeNet. Our final training schedule reduces the learning rate in the last 50 epochs and freezes the backbone, such that only the decoder and prediction head are updated. This design is intended to stabilize late-stage optimization and improve the refinement of boundary-sensitive representations.

To verify its contribution, we compare the proposed strategy with several alternative training schedules: (1) full-network training with a constant learning rate throughout the entire 400 epochs; (2) full-network training with cosine learning-rate decay; (3) applying only the late-stage learning-rate drop without freezing; and (4) applying only late-stage backbone freezing. The quantitative results are reported in Table 6.

**Table 6:** Ablation on different training strategies for HF-EdgeNet on ACDC

Training strategy	Avg.	RV	Myo	LV
Constant LR	91.72	90.47	89.53	95.16
Cosine decay	91.99	90.69	89.83	95.46
LR drop only	91.95	90.68	89.80	95.37
Freeze only	91.80	90.57	89.65	95.17
Ours	**92.14**	**90.83**	**89.84**	**95.74**

The best results are highlighted in bold.

As shown in Table 6, all alternative strategies yield lower performance than the final training scheme adopted in HF-EdgeNet. Using a constant learning rate throughout training gives the weakest result, with an average dice score of 91.72%, suggesting that late-stage refinement is important once the backbone representation becomes relatively stable.

Introducing cosine decay improves the average dice to 91.99%, while using only the late-stage learning-rate drop reaches 91.95%. These results indicate that learning-rate adjustment alone already benefits optimization, but is insufficient to fully exploit the decoder-side structural refinement capability of the proposed framework.

Applying only backbone freezing also leads to a weaker result (91.80%), showing that freezing without a suitable late-stage learning-rate schedule cannot fully stabilize or refine the optimization process. In contrast, the full strategy, which combines learning-rate reduction and backbone freezing while updating only the decoder and prediction head in the final stage, achieves the best performance of 92.14%.

Overall, these results show that the adopted late-stage training strategy is an effective optimization design for HF-EdgeNet, enabling more stable late-stage refinement of boundary-sensitive predictions.

#### Ablation on loss design

We further conduct an ablation study to evaluate the effectiveness of the proposed frequency-aware multi-level supervision objective. As described in training objective: frequency-aware multi-level supervision, the full loss consists of three parts: the basic segmentation loss $L_{\text{seg}}$, the high-frequency structural consistency loss $L_{\text{hf}}$, and the HF-semantic alignment loss $L_{\text{align}}$. These components are designed to supervise region overlap, boundary-sensitive structural consistency, and semantic-edge coordination, respectively.

To verify the contribution of each term, we compare four settings: (1) the baseline supervision using only dice + cross-entropy (CE) loss; (2) adding the high-frequency structural consistency term; (3) adding the HF-semantic alignment term; and (4) the full objective that jointly uses all three losses. The quantitative results are reported in Table 7.

**Table 7:** Ablation on different loss combinations for HF-EdgeNet on ACDC

Setting	$L_{\text{seg}}$	$L_{\text{hf}}$	$L_{\text{align}}$	Avg.
Baseline	✓	×	×	90.75
+ $L_{\text{hf}}$	✓	✓	×	91.93
+ $L_{\text{align}}$	✓	×	✓	91.38
Full objective	✓	✓	✓	**92.14**

The best results are highlighted in bold.

As shown in Table 7, using only the baseline segmentation supervision results in an average dice score of 90.75%. Adding the high-frequency structural consistency loss substantially improves the dice score to 91.93%, confirming that explicitly constraining the predicted masks to match the high-frequency structural responses of the ground truth is beneficial for preserving fine contour details.

When only the HF-semantic alignment loss is added, the dice score increases to 91.38%. This result shows that explicitly encouraging semantic-edge consistency during decoding is also effective, although its contribution is relatively smaller than that of direct high-frequency structural supervision.

The full objective achieves the best performance, reaching 92.14% dice. This indicates that $L_{\text{hf}}$ and $L_{\text{align}}$ are complementary rather than redundant: the former enhances structural consistency at the prediction level, while the latter improves semantic-boundary coordination in the decoder.

Overall, these results demonstrate that the proposed loss design is well motivated. Instead of relying solely on conventional region-based supervision, the full objective explicitly incorporates both structural high-frequency consistency and decoder-side semantic-edge alignment, which together contribute to the superior performance of HF-EdgeNet.

### Parameter analysis

We further investigate the sensitivity of HF-EdgeNet to several key hyperparameters that are closely related to high-frequency modeling and semantic-edge interaction. Specifically, we analyze the effects of (1) the kernel size of the learnable high-frequency operator, (2) the high-frequency injection strength *λ*_inj_, and (3) the depth of the SEB blocks. All experiments are conducted on the ACDC dataset under identical training settings, and dice score (%) is reported.

#### High-frequency kernel size

**Figure 4:**
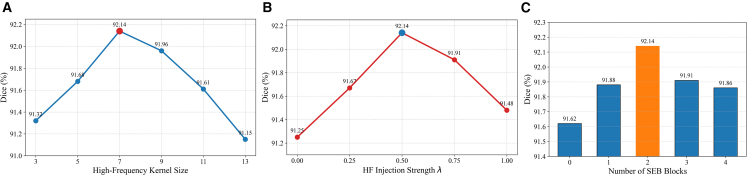
Parameter analysis of HF-EdgeNet on the ACDC dataset (A) Effect of the high-frequency kernel size used in the learnable high-pass operator. The dice score increases when the kernel size is enlarged from 3 to 7 and decreases when the kernel becomes larger, indicating that a moderate kernel size best balances local structural sensitivity and spatial precision. (B) Effect of the high-frequency injection strength λ_inj_ in the HF-EdgeT module. A small λ_inj_ underuses high-frequency structural information, whereas an overly large λ_inj_ may disturb semantic consistency; λ_inj_ = 0.5 achieves the best dice score. (C) Effect of the number of SEB blocks in the decoder. Adding SEB blocks improves segmentation performance by enabling semantic-edge re-alignment, with two SEB blocks achieving the best result; deeper stacking provides no further gain and may introduce redundancy or over-smoothing. Dice score (%) on the ACDC dataset is reported in all images.

Figure 4A illustrates the effect of the kernel size used in the learnable high-frequency extraction operator. As the kernel size increases from 3 to 7, the dice score consistently improves, indicating that moderately enlarged receptive fields facilitate more effective capture of boundary-related high-frequency structures. However, further increasing the kernel size leads to performance degradation, as overly large kernels tend to dilute local structural details and weaken precise boundary localization. Consequently, a kernel size of 7 provides the best trade-off between structural sensitivity and spatial precision.

#### High-frequency injection strength

Figure 4B analyzes the influence of the high-frequency injection strength *λ*_inj_ in the HF-EdgeT module. When *λ*_inj_ is small, high-frequency information is insufficiently emphasized, resulting in suboptimal boundary modeling. As *λ*_inj_ increases, the segmentation performance improves and reaches its peak at *λ*_inj_ = 0.5. Further increasing *λ*_inj_ degrades performance, suggesting that excessive high-frequency injection may interfere with semantic consistency. This observation confirms that a moderate level of high-frequency enhancement is essential for balancing boundary sensitivity and semantic stability.

#### SEB block depth

Figure 4C reports the effect of varying the number of SEB blocks in the decoder. Introducing SEB blocks consistently improves performance compared with the baseline decoder, validating the effectiveness of semantic-edge re-alignment. The best performance is achieved with two SEB blocks, while deeper stacking yields marginal or degraded results. This behavior indicates that excessive semantic-edge interactions may introduce redundancy or over-smoothing, and a shallow SEB configuration is sufficient for effective boundary recovery.

Overall, these results demonstrate that HF-EdgeNet achieves robust performance under a well-balanced configuration of high-frequency modeling strength and semantic-edge interaction depth, further validating the rationality of the proposed design.

## Discussion

The present results indicate that accurate cardiac structure segmentation should not be treated solely as a region-level semantic recognition problem. For thin-walled, low-contrast, and tightly adjacent anatomical structures, segmentation quality depends strongly on whether boundary-sensitive high-frequency information can be effectively modeled and preserved throughout the network. In this study, HF-EdgeNet consistently achieves the best overall dice performance on both ACDC and M&Ms, while also showing favorable boundary-aware geometric results, indicating improved contour fidelity and structural consistency in challenging regions.

**Figure 5:**
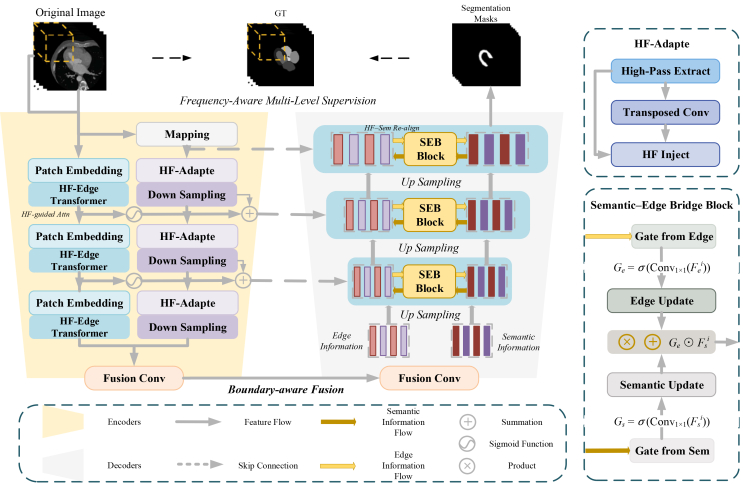
Overall architecture of the proposed HF-EdgeNet. HF-EdgeNet adopts a dual encoder-decoder framework with explicit high-frequency driven modeling The encoder performs high-frequency guided semantic learning via stacked HF-EdgeT blocks and compensates high-frequency loss using HF-Adapte modules before down-sampling. During decoding, SEB blocks re-align semantic features with preserved high-frequency structural cues to enable boundary-consistent reconstruction. The framework integrates high-frequency guided attention, high-frequency preservation, and semantic-edge re-alignment into a closed-loop segmentation pipeline.

**Figure 6:**
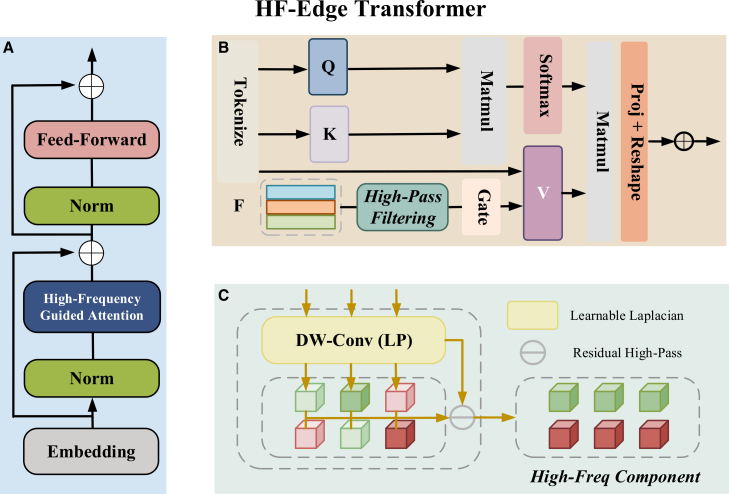
Architecture of the proposed HF-Edge Transformer (A) Overall block formulation, where high-frequency guided attention and feedforward layers are organized in a residual transformer-style structure. (B) High-frequency guided attention, where a learnable high-frequency component is injected into the value branch to enhance boundary-aware feature aggregation. (C) Learnable residual high-pass filtering used for high-frequency extraction. This design allows HF-EdgeT to propagate boundary-sensitive structural information during attention modeling.

A key point of HF-EdgeNet is that high-frequency structural cues are not introduced only as auxiliary supervision, but are integrated into the representation learning pipeline itself. Specifically, HF-EdgeT injects high-frequency guidance into attention modeling, HF-Adapte compensates for structural information loss during down-sampling, and SEB performs explicit semantic-boundary re-alignment during decoding. The overall architecture of HF-EdgeNet and the detailed design of HF-EdgeT are illustrated in Figures 5 and 6, respectively. The ablation studies further support that these components are complementary and jointly contribute to the final performance. Compared with many existing boundary-aware or edge-guided methods, the main distinction of HF-EdgeNet lies in treating high-frequency structural information as a primary modeling factor in feature aggregation, cross-scale preservation, and decoder-side refinement.

### Limitations of the study

Although HF-EdgeNet achieves consistent improvements in cardiac structure segmentation, several method-level limitations still remain. First, the current framework introduces high-frequency modeling in a relatively modular manner, where extraction, compensation, and semantic-boundary re-alignment are designed as separate components. While this design is effective, a more unified and adaptive formulation may further improve the flexibility of structural representation learning. Second, the present method mainly emphasizes local boundary-sensitive cues, whereas the interaction between high-frequency structural details and larger scale anatomical context is still relatively limited. In addition, following common practice in 2D cardiac MRI segmentation, HF-EdgeNet is trained on 2D slices from 3D volumes, which does not explicitly model through-plane spatial continuity.

These limitations also suggest several directions for future work. A promising direction is to develop a more unified frequency-aware modeling framework in which structural cues can be learned and propagated in a more adaptive manner. Another direction is to strengthen the integration of local boundary modeling with global anatomical reasoning, for example through richer volumetric context or stronger shape-aware constraints, so as to further improve structural consistency in challenging cases.

## Resource availability

### Lead contact

Further information and requests for resources should be directed to and will be fulfilled by the lead contact, W.Q. (edwardqinwei@163.com).

### Materials availability

This study did not generate new unique materials.

### Data and code availability

• This study used publicly available cardiac MRI datasets, including ACDC and M&Ms. These datasets are available from their original challenge websites or dataset providers under their respective access policies. Dataset sources and identifiers are listed in the key resources table.
• The custom code generated in this study will be made available by the lead contact, W.Q. (edwardqinwei@163.com), upon reasonable request.
• Any additional information required to reanalyze the data reported in this paper is available from the lead contact, W.Q. (edwardqinwei@163.com), upon reasonable request.

## Acknowledgments

The authors thank the organizers and maintainers of the ACDC and M&Ms datasets for making the cardiac MRI benchmarks publicly available.

## Author contributions

Conceptualization, S.H., H.X., M.Z., H.G., and W.Q.; methodology, S.H., H.X., W.W., and J.Z.; software, S.H. and H.X.; validation, S.H., H.X., W.W., and J.Z.; formal analysis, S.H., H.X., and W.Q.; investigation, S.H., H.X., and W.Q.; writing – original draft, S.H. and H.X.; writing – review and editing, W.W., J.Z., M.Z., H.G., and W.Q.; supervision, M.Z., H.G., and W.Q.; project administration, W.Q. All authors reviewed and approved the final manuscript.

## Declaration of interests

The authors declare no competing interests.

## STAR★Methods

### Key resources table

**Table:** 

REAGENT OR RESOURCE	SOURCE	IDENTIFIER
**Deposited data**		

ACDC cardiac MRI dataset	Bernard et al., 2018	https://www.creatis.insa-lyon.fr/Challenge/acdc/
M&Ms cardiac MRI dataset	Campello et al., 2021	https://www.ub.edu/mnms/

**Software and algorithms**		

Python	Python Software Foundation	https://www.python.org/
PyTorch	PyTorch	https://pytorch.org/
HF-EdgeNet implementation	This paper	Available upon reasonable request to the lead contact

**Other**		

NVIDIA GeForce RTX 4090 GPU	NVIDIA	https://www.nvidia.com/

### Experimental model and study participant details

#### Public datasets

This study used publicly available and de-identified cardiac MRI datasets, including ACDC and M&Ms. No new patients were recruited, and no animal subjects or cell lines were used in this study. Ethical approval and informed consent were handled by the original dataset providers; therefore, additional institutional ethical approval and a new protocol number were not applicable to this secondary analysis of public datasets. Patient demographic information, including age, sex, and ethnicity, was used only when available from the public dataset documentation. Ethnicity information was not available for the datasets used in this study. The influence of sex or gender was not separately analyzed because this study focused on method development and benchmark evaluation using de-identified public datasets. This work was not part of a clinical trial.

### Method details

In this section, we present the proposed HF-EdgeNet framework for cardiac structure segmentation. HF-EdgeNet is a unified high-frequency driven segmentation paradigm that explicitly elevates high-frequency structural cues to a primary modeling factor throughout the entire encoder–decoder pipeline. By integrating high-frequency priors into attention modeling, down-sampling compensation, and decoding re-alignment, HF-EdgeNet forms a closed-loop high-frequency aware segmentation architecture for accurate cardiac structure delineation.

#### Overview of HF-EdgeNet framework

Given an input cardiac image $X\in R^{C\times H\times W}$, HF-EdgeNet adopts a dual encoder–decoder architecture to form a unified high-frequency-driven segmentation framework, as illustrated in Figure 5. The two branches are designed to capture complementary representations, jointly enhancing boundary localization and structural consistency.

Specifically, let E(·) and D(·) denote the encoder and decoder, respectively. The encoder extracts a set of hierarchical feature representations ${\left\{F^{i}\right\}}_{i=1}^{L}$ from the input image, which are progressively fused and decoded to produce the final segmentation prediction:

(Equation 1) $$\hat{Y}=D\left({\left\{F^{i}\right\}}_{i=1}^{L}\right),\text{where}\;{\left\{F^{i}\right\}}_{i=1}^{L}=E\left(X\right)\text{.}$$

##### Encoder with HF-preserving hierarchy

The encoder is organized as a hierarchical multi-stage structure. At each encoding stage, the input features are first transformed into patch-wise embeddings via a *Patch Embedding* layer. The embedded features are then processed by an HF-Edge Transformer (HF-EdgeT) block to perform high-frequency guided semantic modeling, producing boundary-aware semantic representations.

To prevent the degradation of high-frequency structural cues caused by spatial resolution reduction, an HF-Adapte module is explicitly inserted before each down-sampling operation. Given the encoded feature **F**_*i*_, HF-Adapte extracts discriminative high-frequency components and reinjects them into the feature stream to compensate for the high-frequency loss induced by resolution reduction. The compensated features are subsequently down-sampled to form the input of the next stage:

(Equation 2) $$F_{i+1}=\text{Down}\left(A_{i}\left(F_{i}\right)\right),i=1,\ldots ,L-1\text{,}$$

where $A_{i}\left(\cdot \right)$ denotes the HF-Adapte module at the *i*-th stage.

##### Decoder with HF–semantic re-alignment

The decoder progressively reconstructs high-resolution representations through a sequence of up-sampling stages. At each decoding level, the up-sampled semantic features are fed into a Semantic–Edge Bridge (SEB) block, where preserved high-frequency features propagated from the encoder are explicitly fused to perform HF–semantic re-alignment. This bidirectional interaction enables boundary-aware semantic recovery and suppresses spurious responses caused by scale transitions:

(Equation 3) $${\tilde{F}}_{j-1}=B_{j}\left(\text{Up}\left({\tilde{F}}_{j}\right),H_{j-1}\right),j=L,\ldots ,2\text{,}$$

where $\text{Up}\left({\tilde{F}}_{j}\right)$ denotes the up-sampled semantic feature from the *j*-th decoding stage, **H**_*j*-1_ denotes the high-frequency structural feature preserved by HF-Adapte at the corresponding encoding stage, and $B_{j}\left(\cdot \right)$ denotes the SEB-based bridging operator at stage *j*.

Finally, the re-aligned features are aggregated by a boundary-aware fusion head to generate the segmentation prediction:

(Equation 4) $$\hat{Y}=\text{Head}\left({\tilde{F}}_{1}\right)\text{.}$$

Through the hierarchical integration of HF-guided attention modeling (HF-EdgeT), HF-preserving down-sampling compensation (HF-Adapte), and HF–semantic re-alignment decoding (SEB), HF-EdgeNet forms a closed-loop high-frequency driven segmentation paradigm for accurate cardiac structure delineation.

#### HF-EdgeT: HF-guided attention modeling

Cardiac structure segmentation is inherently boundary-sensitive, where discriminative anatomical cues are often encoded in high-frequency structures around tissue transitions. However, existing Transformer-based segmentation backbones usually perform attention aggregation mainly according to region-level semantic similarity, which may under-emphasize fine boundary variations. Motivated by this observation, we propose an HF-Edge Transformer (HF-EdgeT) that explicitly injects a learnable high-frequency field into the value pathway, thereby enabling boundary-aware semantic aggregation.

As illustrated in Figure 6A, HF-EdgeT follows a standard pre-norm residual formulation, while replacing the vanilla attention with a **High-Frequency Guided Attention** unit. Given an input feature map $F\in R^{C\times H\times W}$, where *H* and *W* denote the token-grid height and width after patch embedding or window partitioning, we first serialize the spatial feature into a token sequence:

(Equation 5) $$X=S\left(F\right)\in R^{N\times C},N=H\times W\text{,}$$

where S(·) denotes the serialization operator that flattens the spatial dimensions into a token sequence. For simplicity, the batch dimension is omitted in this subsection, and the attention formulation is written in single-head form.

The query, key, and value embeddings are then computed in token space as

(Equation 6) $$Q=XW_{Q},K=XW_{K},V=XW_{V}\text{,}$$

where $W_{Q},W_{K}\in R^{C\times d}$ and $W_{V}\in R^{C\times d_{v}}$ are learnable projection matrices. Accordingly, $Q,K\in R^{N\times d}$ and $V\in R^{N\times d_{v}}$. Here and hereafter, juxtaposition denotes matrix multiplication.

##### Learnable high-frequency extraction

To obtain the structural high-frequency field, we adopt a learnable residual high-pass operator, as illustrated in Figure 6C. Specifically, a depth-wise low-pass convolution is first applied to the spatial feature, and the residual high-frequency response is computed by subtraction:

(Equation 7) $$F_{\text{hf}}=F-{\text{DWConv}}_{\text{LP}}\left(F\right)\text{,}$$

where $F_{\text{hf}}\in R^{C\times H\times W}$ denotes the learnable high-frequency structural field. This residual formulation explicitly emphasizes boundary-related responses while remaining fully learnable and data-adaptive.

##### High-frequency injection into value

Instead of modifying the attention logits, we inject the high-frequency field into the *value* branch to explicitly enrich the propagated content with boundary-sensitive structures. We first project **F**_hf_ to the value dimension and generate a gated structural term in the spatial domain:

(Equation 8) $$\Delta \bar{V}={\text{Conv}}_{1\times 1}\left(F_{\text{hf}}\right),{\bar{G}}_{h}=\sigma \left({\text{Conv}}_{1\times 1}\left(F_{\text{hf}}\right)\right)\text{,}$$

where $\Delta \bar{V},{\bar{G}}_{h}\in R^{d_{v}\times H\times W}$. We then serialize these spatial tensors into token space:

(Equation 9) $$\Delta V=S\left(\Delta \bar{V}\right)\in R^{N\times d_{v}},G_{h}=S\left({\bar{G}}_{h}\right)\in R^{N\times d_{v}}\text{.}$$

The high-frequency enhanced value is finally constructed as

(Equation 10) $$\tilde{V}=V+\lambda_{\text{inj}}\left(G_{h}⊙\Delta V\right)\text{,}$$

where *λ*_inj_ denotes the high-frequency injection strength, + denotes element-wise addition, ⊙ denotes element-wise multiplication, and *σ*(·) denotes the sigmoid activation.

Here, **G**_*h*_ serves as a content-adaptive modulation factor that controls the token-wise and channel-wise amplitude of high-frequency injection, while *λ*_inj_ provides a global scaling coefficient for the overall injection strength. As a result, the model does not indiscriminately propagate all high-frequency responses; instead, boundary-consistent structural cues can be selectively enhanced, while weak or structurally irrelevant activations are attenuated. From a feature-flow perspective, the gate determines where the injected structural response should be emphasized, whereas *λ*_inj_ controls how strongly the high-frequency branch perturbs the propagated value content. From an optimization perspective, this formulation also stabilizes training by preventing overly weak or overly aggressive high-frequency injection. In this way, high-frequency structural cues are embedded into the value content in a controlled rather than unconditional manner.

##### HF-guided attention aggregation

Attention weights are computed from **Q** and **K**, while the enhanced value $\tilde{V}$ is used for aggregation:

(Equation 11) $$A=\text{Softmax}\left(\frac{QK^{⊤}}{\sqrt{d}}\right),X^{\prime }=A\tilde{V}\text{,}$$

where $A\in R^{N\times N}$ is the attention map and $X^{\prime }\in R^{N\times d_{v}}$ is the attended token representation.

To restore the spatial feature format, we project the attended tokens back to the original channel dimension and reshape them to the token grid:

(Equation 12) $$F^{\prime }=R\left(X^{\prime }W_{O}\right)\in R^{C\times H\times W}\text{,}$$

where $W_{O}\in R^{d_{v}\times C}$ is the output projection, and R(·) denotes the inverse reshape operator of S(·).

Finally, the output of the High-Frequency Guided Attention is added back to the input via a residual connection:

(Equation 13) $$F_{\text{out}}=F+F^{\prime }\text{.}$$

Therefore, HF-EdgeT performs attention on serialized token features while explicitly injecting a learnable high-frequency field into the value pathway. As summarized in Figure 6B, the overall procedure can be abstracted into three steps: tokenization for query/key generation, high-frequency-guided value enhancement, and projection/reshape after attention aggregation. Through this design, HF-EdgeT explicitly propagates boundary-sensitive structural information during attention modeling, yielding boundary-aware semantic representations that are beneficial for accurate cardiac structure segmentation.

#### HF-Adapte: High-frequency compensation for down-sampling

Progressive down-sampling operations are widely adopted in encoder–decoder architectures to enlarge receptive fields and reduce computational cost. However, such operations inevitably attenuate high-frequency components that are critical for boundary delineation in cardiac structure segmentation, leading to the loss of thin and low-contrast anatomical details. To mitigate this degradation, we propose an HF-Adapte module that explicitly compensates for high-frequency information loss induced by down-sampling.

Given an input feature map $F_{i}\in R^{C_{i}\times H_{i}\times W_{i}}$ at the *i*-th encoding stage, we first extract its high-frequency representation:

(Equation 14) $$H_{i}=H\left(F_{i}\right)\text{,}$$

where H(·) denotes the same learnable high-pass filtering operator as used in HF-EdgeT.

The extracted high-frequency features are projected into a gating map:

(Equation 15) $$G_{i}=\sigma \left(H_{i}W_{i}\right)\text{,}$$

where **W**_*i*_ denotes a learnable unit convolution and *σ*(·) denotes the sigmoid function. Meanwhile, the original features are down-sampled:

(Equation 16) $$F_{i}^{↓}=\text{Down}\left(F_{i}\right)\text{.}$$

To compensate for the attenuated high-frequency components, the gated and down-sampled high-frequency features are reinjected into the down-sampled features via a residual formulation:

(Equation 17) $$F_{i+1}=F_{i}^{↓}⊕\left(G_{i}⊙\text{Down}\left(H_{i}\right)\right)\text{,}$$

where ⊕ and ⊙ denote element-wise addition and multiplication, respectively.

Through the gated residual compensation, HF-Adapte explicitly preserves discriminative high-frequency structural cues across scales, alleviating the degradation of thin and low-contrast anatomical boundaries during progressive down-sampling.

#### SEB block: HF–semantic Re-alignment

Although skip connections can preserve and transfer high-resolution shallow features from the encoder to the decoder, they mainly provide implicit feature fusion rather than explicit semantic–boundary calibration. After repeated down-sampling and up-sampling, the decoder feature becomes increasingly semantic and context-rich, but may also suffer from boundary blurring or spatial drift. Meanwhile, the encoder-transferred high-frequency feature preserves fine structural responses, yet these responses are not necessarily aligned with the current semantic transition field and may also contain structurally irrelevant activations. Therefore, the decoding stage still requires an explicit mechanism to re-align restored semantic representations with boundary-sensitive structural cues.

To this end, we propose a **Semantic–Edge Bridge (SEB) block**, which performs bidirectional calibration between the up-sampled semantic feature and the edge-aware high-frequency feature at the same decoding scale. Instead of passively fusing features through a standard skip pathway, SEB explicitly models how boundary-sensitive responses should refine semantic transitions, while semantic context simultaneously suppresses structurally irrelevant high-frequency activations.

Let $F_{s}^{i}\in R^{C_{s}\times H_{i}\times W_{i}}$ denote the up-sampled semantic feature at the *i*-th decoding stage, and let $F_{e}^{i}\in R^{C_{e}\times H_{i}\times W_{i}}$ denote the edge-aware high-frequency feature propagated from the corresponding encoder scale. For notational simplicity, the spatial sizes of the two branches are assumed to be aligned at the current decoding stage.

SEB first generates a structure-aware gate from the edge-aware feature:

(Equation 18) $$G_{e}^{i}=\sigma \left({\text{Conv}}_{1\times 1}\left(F_{e}^{i}\right)\right)\text{,}$$

where $G_{e}^{i}\in R^{C_{s}\times H_{i}\times W_{i}}$ is used to modulate the semantic branch. The edge-guided semantic refinement is then written as

(Equation 19) $${\tilde{F}}_{s}^{i}=F_{s}^{i}+G_{e}^{i}⊙F_{s}^{i}\text{,}$$

where ⊙ denotes element-wise multiplication. Through this operation, edge-aware responses explicitly sharpen semantic transitions and enhance boundary-sensitive activations.

Meanwhile, the semantic feature provides region-level context to constrain the edge-aware branch and suppress spurious high-frequency responses:

(Equation 20) $$G_{s}^{i}=\sigma \left({\text{Conv}}_{1\times 1}\left(F_{s}^{i}\right)\right)\text{,}$$

where $G_{s}^{i}\in R^{C_{e}\times H_{i}\times W_{i}}$ is used to recalibrate the edge-aware feature. The semantic-guided edge refinement is formulated as

(Equation 21) $${\tilde{F}}_{e}^{i}=F_{e}^{i}+G_{s}^{i}⊙F_{e}^{i}\text{.}$$

This step helps preserve anatomically meaningful boundary responses while suppressing structurally irrelevant or noisy high-frequency activations.

Finally, the re-aligned semantic and edge-aware features are fused to produce the SEB output:

(Equation 22) $$F_{\text{out}}^{i}={\text{Conv}}_{1\times 1}\left(\left[{\tilde{F}}_{s}^{i},{\tilde{F}}_{e}^{i}\right]\right)\text{,}$$

where [·,·] denotes channel-wise concatenation.

Compared with a conventional skip connection that mainly forwards shallow encoder features, SEB explicitly performs bidirectional semantic–boundary re-alignment at the decoding stage. By reconstructing semantic representations under the guidance of edge-aware structural cues, while simultaneously filtering the edge branch with semantic context, the proposed SEB block effectively alleviates boundary drifting and facilitates accurate recovery of thin and low-contrast anatomical structures.

Through the synergistic integration of high-frequency guided attention modeling (HF-EdgeT), high-frequency compensation for down-sampling (HF-Adapte), and high-frequency–semantic re-alignment during decoding (SEB block), HF-EdgeNet forms a unified high-frequency driven segmentation framework. This closed-loop design explicitly models, preserves, and exploits high-frequency structural cues throughout the entire encoder–decoder pipeline, enabling boundary-aware semantic representation learning and accurate recovery of thin and low-contrast anatomical structures. As a result, HF-EdgeNet provides a principled and effective paradigm for precise cardiac structure delineation.

#### Training objective: Frequency-aware multi-level supervision

HF-EdgeNet is optimized with a frequency-aware multi-level supervision objective, which explicitly regularizes both region-level semantics and boundary-sensitive high-frequency structures. The overall loss is formulated as

(Equation 23) $$L=L_{\text{seg}}+\lambda_{\text{hf}}\;L_{\text{hf}}+\lambda_{\text{align}}\;L_{\text{align}}\text{,}$$

where *λ*_hf_ and *λ*_align_ balance the contributions of high-frequency structure supervision and HF–semantic alignment.

(1) **Segmentation loss** Let $\hat{Y}\in {\left[0,1\right]}^{C\times H\times W}$ be the predicted probability map and **Y**∈{0,1}^*C*×*H*×*W*^ be the one-hot ground-truth label. We adopt a standard Dice+CE objective:

(Equation 24) $$L_{\text{seg}}=L_{\text{ce}}\left(\hat{Y},Y\right)+L_{\text{dice}}\left(\hat{Y},Y\right)\text{,}$$

with

(Equation 25) $$L_{\text{ce}}=-\frac{1}{N}\sum_{c=1}^{C}\sum_{u\in \Omega }Y_{c}\left(u\right)\log\left({\hat{Y}}_{c}\left(u\right)+\epsilon \right)\text{,}$$

(Equation 26) $$L_{\text{dice}}=1-\frac{1}{C}\sum_{c=1}^{C}\frac{2\sum_{u\in \Omega }{\hat{Y}}_{c}\left(u\right)Y_{c}\left(u\right)+\epsilon }{\sum_{u\in \Omega }{\hat{Y}}_{c}{\left(u\right)}^{2}+\sum_{u\in \Omega }Y_{c}{\left(u\right)}^{2}+\epsilon }\text{,}$$

where Ω denotes the spatial domain, *u* indexes a spatial location, *N* = |Ω|, and ϵ is a small constant.

(2) **High-frequency structural consistency loss** To explicitly supervise boundary-sensitive structures, we enforce that the predicted segmentation exhibits consistent high-frequency responses with the ground truth under the same learnable high-pass operator. Specifically, for a multi-scale pyramid ${\left\{s\right\}}_{s=1}^{S}$, we down-sample $\hat{Y}$ and **Y** to the *s*-th scale, denoted by ${\hat{Y}}^{\left(s\right)}$ and **Y**^(*s*)^. Their high-frequency responses are computed via a residual high-pass operator:

(Equation 27) $$H\left(Z\right)=Z-{\text{DWConv}}_{\text{LP}}\left(Z\right)\text{,}$$

where DWConv_LP_(·) denotes a learnable depth-wise low-pass convolution shared across the framework. The HF consistency loss is then defined as

(Equation 28) $$L_{\text{hf}}=\sum_{s=1}^{S}\alpha_{s}\;{\left\|H\left({\hat{Y}}^{\left(s\right)}\right)-\text{stopgrad}\left(H\left(Y^{\left(s\right)}\right)\right)\right\|}_{1}\text{,}$$

where *α*_*s*_ is a scale weight and ‖·‖_1_ denotes the element-wise $l_{1}$ norm averaged over all spatial locations and channels. Here, stopgrad(·) denotes the stop-gradient operator, which treats the target high-frequency response as a fixed reference during backpropagation. This design stabilizes the auxiliary supervision by preventing the reference branch from becoming a simultaneously moving target.

(3) **HF–semantic alignment loss (SEB regularization)** SEB aims to re-align semantic transition responses with edge-aware high-frequency cues. To explicitly encourage boundary-consistent reconstruction, we regularize the alignment between the semantic transition field and the high-frequency structural field.

Let $F_{s}^{i}$ and $F_{e}^{i}$ denote the semantic feature and the edge-aware feature fed into SEB at decoding stage *i*. They are first mapped into single-channel attention maps:

(Equation 29) $$S^{i}=\sigma \left({\text{Conv}}_{\text{unit}}\left(F_{s}^{i}\right)\right),E^{i}=\sigma \left({\text{Conv}}_{\text{unit}}\left(F_{e}^{i}\right)\right)\text{.}$$

We then compute their spatial gradient magnitudes ∇(·) (implemented using fixed Sobel or Prewitt operators) and enforce consistency:

(Equation 30) $$L_{\text{align}}=\sum_{i=1}^{I}\beta_{i}\;{\left\|\left|\nabla S^{i}\right|-\text{stopgrad}\left(\left|\nabla E^{i}\right|\right)\right\|}_{1}\text{,}$$

where *β*_*i*_ weights different decoding stages. Similarly, the stop-gradient operator is used here to keep the edge-aware structural target relatively stable when supervising the semantic transition field, thereby avoiding unstable co-adaptation between the two sides of the auxiliary alignment objective.

##### Implementation notes

Unless otherwise specified, we set *S* = 3 and choose *α*_*s*_ = 2^-(*s*−1)^ to emphasize high-resolution supervision. We set *λ*_hf_ = 0.5 and *λ*_align_ = 0.5 in all experiments. The stop-gradient operator is only used in the auxiliary consistency terms above, while the main segmentation pathway remains optimized end-to-end in the standard manner.

### Quantification and statistical analysis

Segmentation performance was quantified using Dice score, Precision, Recall, HD95, and ASSD. All evaluation metrics were computed at the patient level. For paired comparisons of average Dice scores, two-sided Wilcoxon signed-rank tests were performed on paired test cases. No statistical method was used to predetermine sample size.
